# Supplementary material for: Benchmarking computational decontamination of ambient RNA
Source: Front Bioinform. 2026 Aug 19;6:1844838. doi: 10.3389/fbinf.2026.1844838 (PMC13533975; doi:10.3389/fbinf.2026.1844838)
Supplement: Supplementary file 1 [file DataSheet1.pdf]

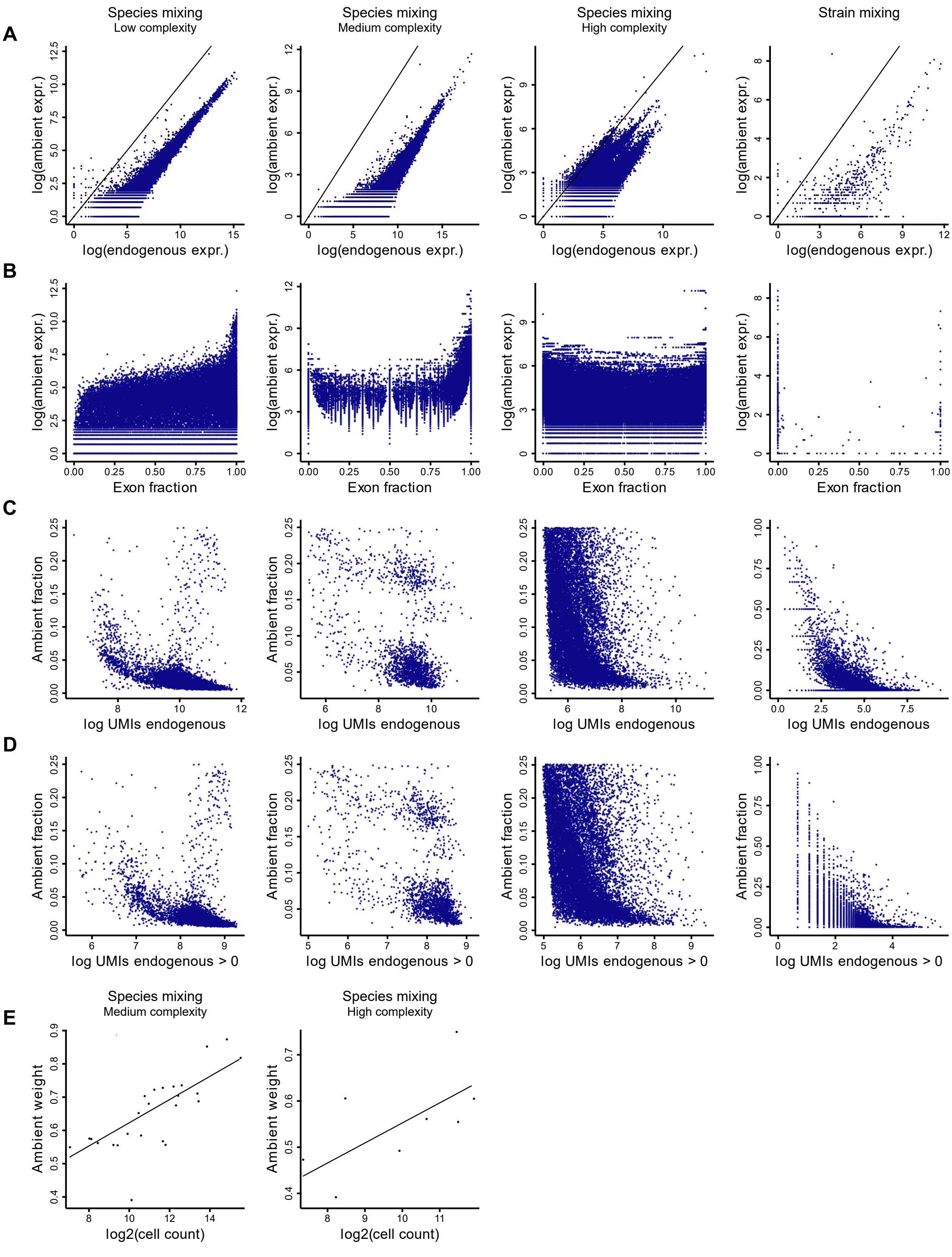

Supplemental Figure S1 (related to Figure 2). A) Scatterplot showing the log ambient and endogenous expression level per gene. **B)** Scatterplot showing the log ambient expression level and fraction of UMIs derived from exons per gene. **C)** Scatterplot showing the log total UMI count and the fraction of UMIs derived from ambient RNA per cell. **D)** Scatterplot showing the log total feature count and the fraction of UMIs derived from ambient RNA per cell. **E)** Scatterplot showing the ambient weight (average regression coefficient between gene expression profile and ambient profile) for clusters and the number of cells in the cluster for the indicated datasets.

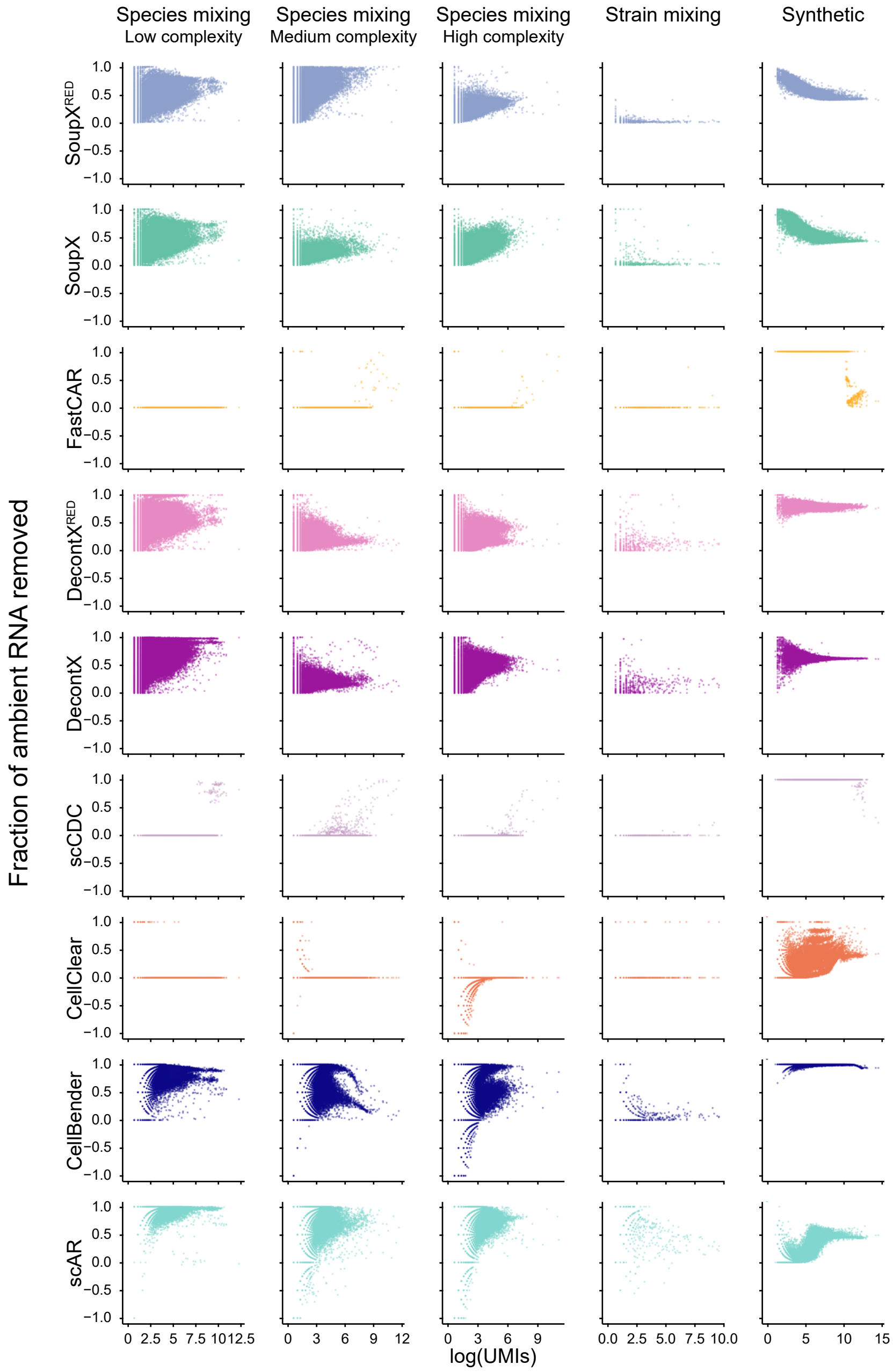

Supplemental Figure S2 (related to Figure 3). Scatterplot showing the log endogenous expression and the fraction of ambient RNA removed for the indicated methods in the indicated datasets.

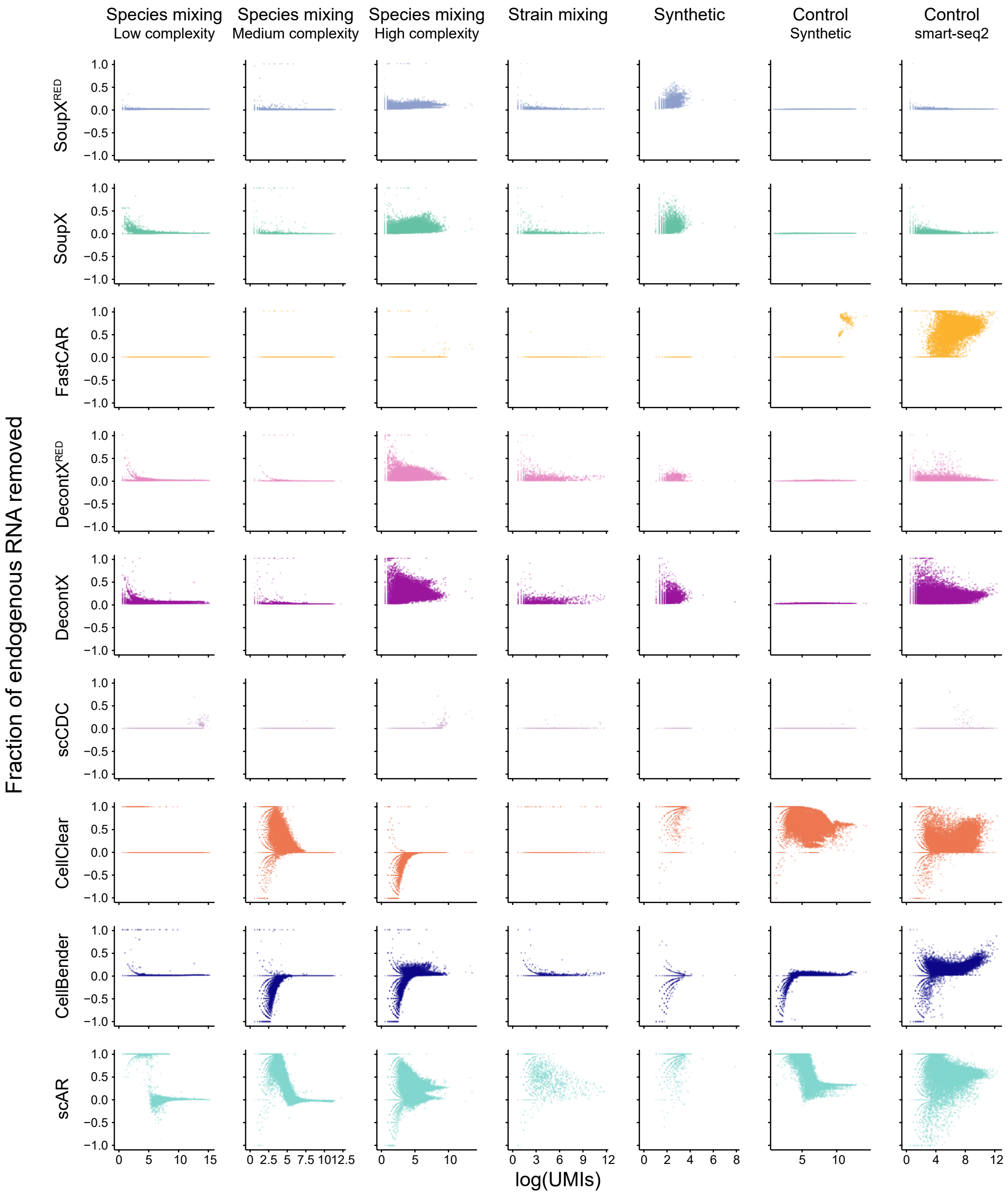

Supplemental Figure S3 (related to Figure 3). Scatterplot showing the log endogenous expression and the fraction of endogenous RNA removed for the indicated methods in the indicated datasets.

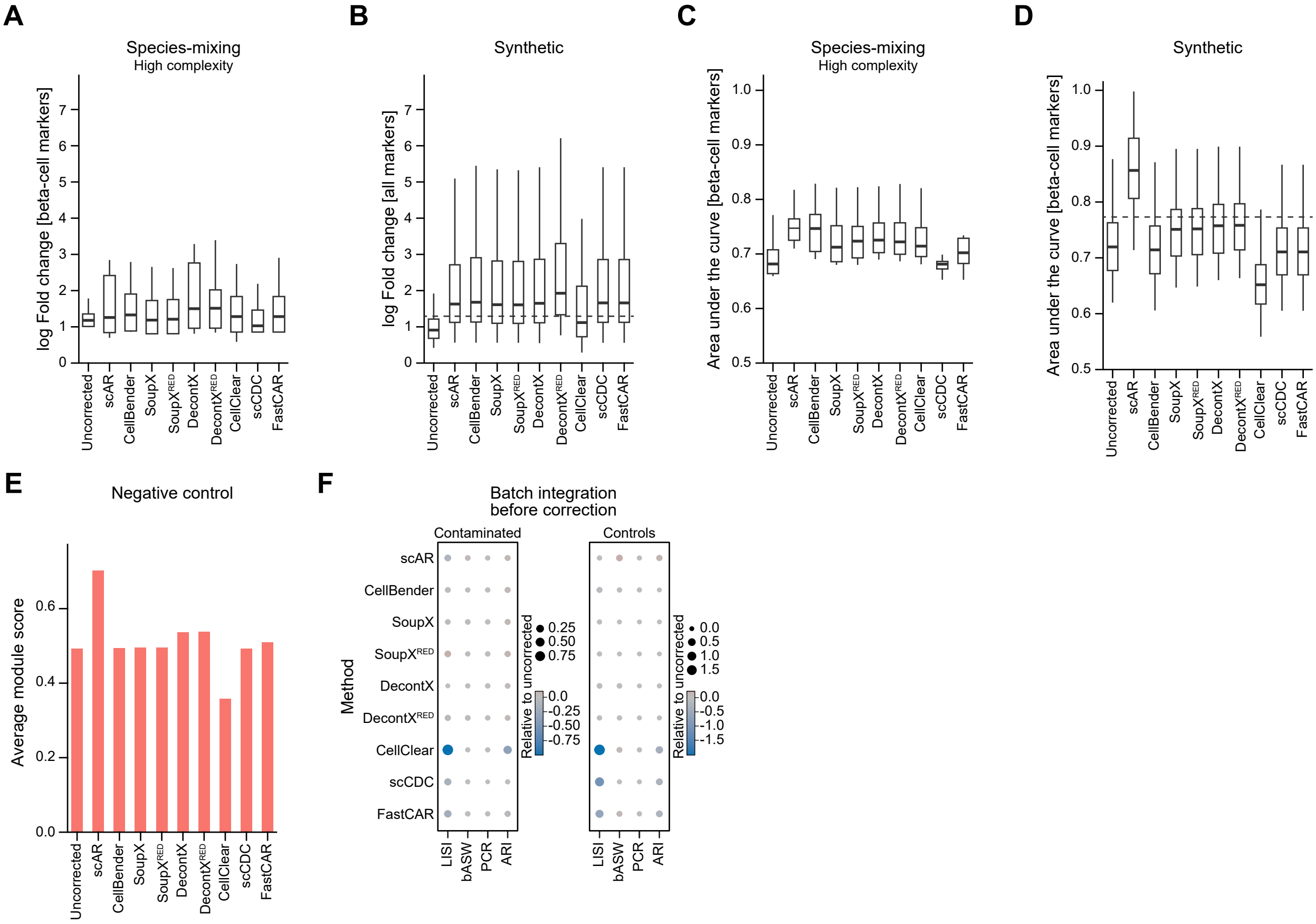

Supplemental Figure S4 (related to Figure 4). A) Barplot showing the log2 fold change of beta-cell marker genes in beta-cells compared to all other cells in high complexity species-mixing dataset. **B)** Barplot showing the log2 fold change of marker genes in the synthetic dataset. **C)** Barplot showing the area under the curve of beta-cell marker genes in beta-cells compared to all other cells in high complexity species-mixing dataset. **D)** Barplot showing the area under the curve of marker genes in the synthetic dataset. **E)** Barplot showing average module scores for marker genes defined in the ground truth dataset from Figure 4A in the same sample without ambient RNA contamination**. F)** Dotplots showing the average of the indicated metrics relative to the uncorrected data for contaminated or control datasets for batch integration prior to integration.
